# Investigating organizational quality improvement systems, patient empowerment, organizational culture, professional involvement and the quality of care in European hospitals: the 'Deepening our Understanding of Quality Improvement in Europe (DUQuE)' project

**DOI:** 10.1186/1472-6963-10-281

**Published:** 2010-09-24

**Authors:** Oliver Groene, Niek Klazinga, Cordula Wagner, Onyebuchi A Arah, Andrew Thompson, Charles Bruneau, Rosa Suñol

**Affiliations:** 1 Avedis Donabedian University Institute, Autonomous University of Barcelona, CIBER Epidemiology and Public Health, Barcelona, Spain; 2 Department of Social Medicine, Academic Medical Centre, Amsterdam, the Netherlands; 3 Netherlands Institute of Health Services Research (NIVEL), Utrecht, the Netherlands; 4 Department of Epidemiology, School of Public Health, University of California, Los Angeles (UCLA); and the Center for Health Policy Research, UCLA, Los Angeles, USA; 5 School of Social and Political Science, University of Edinburgh, Edinburgh, UK; 6 Haute Autorité de la Sante, Paris, France

## Abstract

**Background:**

Hospitals in European countries apply a wide range of quality improvement strategies. Knowledge of the effectiveness of these strategies, implemented as part of an overall hospital quality improvement system, is limited.

**Methods/Design:**

We propose to study the relationships among organisational quality improvement systems, patient empowerment, organisational culture, professionals' involvement with the quality of hospital care, including clinical effectiveness, patient safety and patient involvement. We will employ a cross-sectional, multi-level study design in which patient-level measurements are nested in hospital departments, which are in turn nested in hospitals in different EU countries. Mixed methods will be used for data collection, measurement and analysis. Hospital/care pathway level constructs that will be assessed include external pressure, hospital governance, quality improvement system, patient empowerment in quality improvement, organisational culture and professional involvement. These constructs will be assessed using questionnaires. Patient-level constructs include clinical effectiveness, patient safety and patient involvement, and will be assessed using audit of patient records, routine data and patient surveys. For the assessment of hospital and pathway level constructs we will collect data from randomly selected hospitals in eight countries. For a sample of hospitals in each country we will carry out additional data collection at patient-level related to four conditions (stroke, acute myocardial infarction, hip fracture and delivery). In addition, structural components of quality improvement systems will be assessed using visits by experienced external assessors. Data analysis will include descriptive statistics and graphical representations and methods for data reduction, classification techniques and psychometric analysis, before moving to bi-variate and multivariate analysis. The latter will be conducted at hospital and multilevel. In addition, we will apply sophisticated methodological elements such as the use of causal diagrams, outcome modelling, double robust estimation and detailed sensitivity analysis or multiple bias analyses to assess the impact of the various sources of bias.

**Discussion:**

Products of the project will include a catalogue of instruments and tools that can be used to build departmental or hospital quality and safety programme and an appraisal scheme to assess the maturity of the quality improvement system for use by hospitals and by purchasers to contract hospitals.

## Background

A substantial amount of research has been carried out in the last 30 years on assessing and improving the quality of health care. One of the shifts in focus documented in this literature is the change from research questions such as "how can quality be measured?" to "how can quality be improved?" [1-4]. Despite considerable progress in answering both questions and the widespread application of quality strategies (such as accreditation systems, organisational quality management programmes, audit, patient safety systems, clinical practice guidelines, performance indicators and systems for getting patient views), quality and safety problems persist and the debate on how to accelerate and sustain quality improvement is more relevant than ever [5-9]. In response to this debate a new research line related to the effectiveness of quality improvement emerged from the quality field in the last 10 to 15 years. This led to research questions such as "does quality improvement lead to better quality of care?", "which quality tools are most effective?", "how can various quality tools be integrated into a context-sensitive quality and safety improvement programme?" or "what factors impact on the implementation of quality strategies at hospital level?" [10-13]. These questions are of high relevance from the perspective of individual health care professionals and hospitals, which on the one hand have to comply with existing legislation and statutory regulation of quality improvement, but on the other hand have significant sovereignty to select from a range of quality tools to target organisation-specific quality and safety problems. Professionals and hospitals alike are investing a lot of time and energy in quality improvement efforts, although scientific evaluation of the various approaches, and thus a systematic effort to enhance knowledge and learning, is still limited.

## Research on assessing the impact of quality improvement

Within the European Union, a range of research projects has addressed the evaluation of quality strategies at hospital level. Among the first was the Concerted Action Programme on Quality Assurance in Hospitals (COMAC/HSR/QA), executed between 1990 and 1997 in up to 15 European countries [14]. It identified different situational and operational factors at the national, hospital and topic level, that seemed to influence the effectiveness of strategies for the implementation of quality assurance. The EU BIOMED-funded ExPeRT project, carried out from 1996-1999, aimed at evaluating the use and development of external peer review models and to identify where the main models are used in EU Member States. The project identified four principal models in use in Europe: ISO, EFQM, peer review and accreditation, and demonstrated that, in principle, convergence of the four main models in order to gain from each model's key strengths is feasible [15-17]. Another EU project, the ENQuaL network, was set up as a collaboration between research experts on quality assessment and quality management in European countries. The aim of the network was to facilitate the exchange of knowledge and expertise among European countries. As part of the working plan, a questionnaire for the evaluation of quality and safety management in hospitals was developed [11]. This questionnaire has been used to compare the amount of quality improvement activities between countries [18], as well as considering the longitudinal development of quality improvement systems within one country [19].

The recently EU funded project on Methods of Assessing Response to Quality Improvement Strategies (MARQuIS) added a new focus to the existing literature. This project intended to assess the value of different quality strategies in European hospitals and to provide the needed information for countries when contracting care for patients moving across borders. The project demonstrated substantial variability in the development of hospitals' quality improvement systems both within and between countries. It also indicated that no single strategy, but rather the combination of quality improvement strategies, was associated with the accomplishment of favourable hospital outputs. As an exception, external pressure appeared to be consistently associated with the implementation of quality improvement strategies at hospital level [20-24].

The use of surveys of culture and organisation to assess associations with hospital quality was spearheaded since the early 1990 s by Shortell and colleagues in the USA [25-27]. Some of these studies, based on non-randomised designs, produced tentative evidence of the impact of continuous quality improvement on clinical practice, which interacts with physician involvement, individual practitioner's feedback and supportive organisational culture. However, randomised studies were not able to demonstrate this impact on clinical outcomes. Recent work addressed the impact of organisational quality improvement strategies on hospital patient safety and quality and supported the proposition that the scope of quality improvement implementation in hospitals is significantly associated with hospital-level quality indicators, even though the directions of the associations are not always systematically positive [12,13]. The authors concluded that the successful clinical application of quality improvement actions depends largely on a supportive regulatory and competitive environment, its alignment with financial incentives and with an organisational leadership that is committed to integrating all aspects of the work.

More recently, research also addresses evaluation and impact of hospital accreditation programmes [28]. In a recent systematic review of the accreditation literature, Greenfield and Braithwaite categorised the impact of accreditation under ten topics: professions' attitudes to accreditation, promotion of change, organisational impact, financial impact, quality measures, professional development programme assessment, 'consumer' views or patient satisfaction, public disclosure, and surveyor issues [7]. They found that, in the studies included, accreditation was consistently associated with promoting change and professional development. They also found that the impact on professions' attitudes to accreditation, organisational impact, financial impact, quality measures and programme assessment was inconsistent in the literature. A recent analytical study by the same authors corroborated these findings and demonstrated that accreditation predicts leadership and cultural characteristics, but was not related with organisational climate or 'consumer' participation [29].

In summary, the evidence on the factors associated with the uptake of hospitals of quality improvement, the impact of quality improvement systems on patient-level outcomes, and the association of quality improvement systems with constructs such as professional involvement, patient empowerment and organizational culture, is limited. These links will be explored further in the DUQUE research project.

## Methods/Design

### Overall design

**Figure 1:**
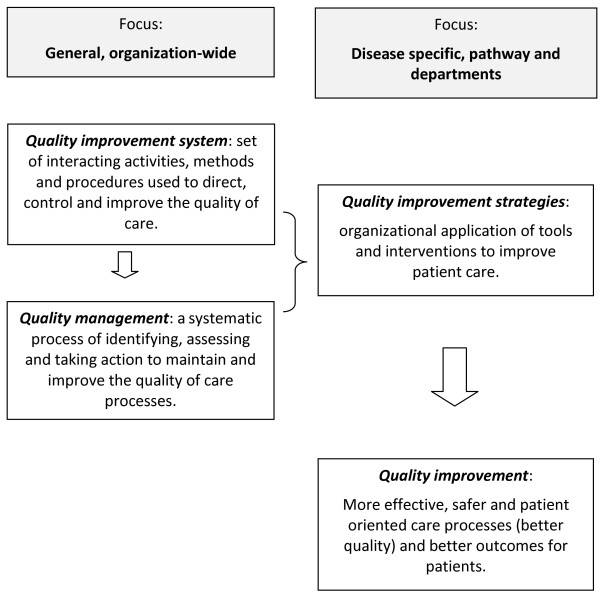
Conceptualization of quality improvement at hospital, pathway and patient level

This project builds on the work developed to understand the impact of the implementation of different systems, such as clinical practice guidelines, and aims to address their global effects integrated in an organisational quality improvement system. Given that quality improvement research is inherently complex, in part due to its interdisciplinary nature and the ethical, methodological and technical limitations in carrying out large-scale experimental studies, different levels of organisational analysis need to be distinguished. Consequently, this project will use mixed methods in data collection, measurement and analysis. The different approaches and levels of quality improvement within the hospital will be conceptualized as follows (Figure 1).

The overall aim of this project is to study to what extent organisational quality improvement systems, organisational culture, professional involvement and patient empowerment are related to the quality of hospital care, assessed in terms of clinical effectiveness, patient safety and patient involvement in a sample of European hospitals.

Specific objectives to be pursued are the following:

**- 1: **To develop a "maturity classification model" for the assessment of organisational quality improvement systems in EU hospitals.

**- 2: **To investigate associations between the maturity of quality improvement systems and measures of organisational culture, professional involvement and patient empowerment (at hospital level).

**- 3: **To investigate associations between the maturity of quality improvement systems and measures of clinical effectiveness, patient safety and patient involvement (at patient and departmental level).

**- 4: **To identify factors influencing the uptake of quality improvement activities by hospitals, including external pressure as enforced by accreditation, certification or external assessment programmes.

**Figure 2:**
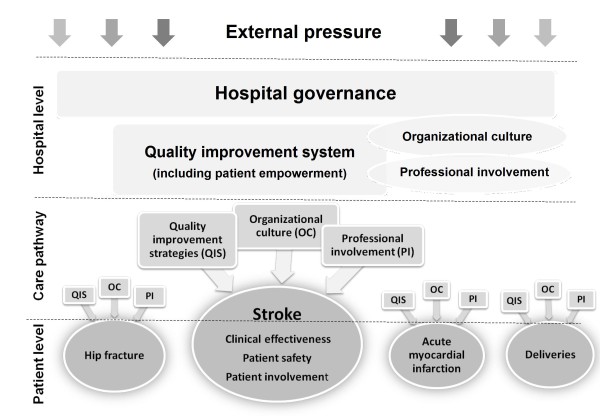
Conceptual model for the multi-level DUQuE study: constructs and hypothesized relationships

Data will be collected cross-sectionally at hospital, care pathway, professional and patient level. We will employ a multi-level study design in which patient-level measurements are nested in hospital departments, which are in turn nested in hospitals in different EU countries. The objectives of the project and the different constructs (explained below) that will be assessed within the DUQuE project can be illustrated as follows (Figure 2).

### Setting and sampling

We will collect data in eight countries with a mix of health and hospital system financing and organisation in different geographical areas in Europe. Recruitment among hospitals will be made in each country randomly using criteria defined by the scientific group. Random lists of hospitals will be drawn to compose both the sample of initial hospitals and of substitute hospitals. If a hospital in the initial sample declines the invitation to participate, substitute hospitals will be added, based on the organisational sampling criteria (ownership, type of hospitals) in order to maintain the representativeness of the sample at country level.

Hospital-wide constructs will be assessed among the selected hospitals in each of the eight participating countries. In each country, for a sub-sample of these hospitals we will carry out additional data collection at the patient-level. Patient-level data will be collected for four conditions, namely stroke, acute myocardial infarction, hip fracture and child delivery. These conditions were chosen based on the following criteria: high financial volume, high prevalence of the condition, existence of validated measures for its assessment, high variability of process and outcomes in the literature, and coverage of different types of patients and specialists. For each condition, randomly selected patient records will be reviewed. Given the multi-level design of the project, the number of patient records to be reviewed is substantially lower than in a traditional design. Power calculations were performed and will be refined, once the measures have been selected, to determine the discriminatory power of the measures.

### Measure development

#### Hospital and pathway level measures

For this project, wherever possible, existing instruments will be used rather than developing new measures. General criteria for selection of measures are the following: scientific properties (e.g. validity, reliability, association with outcomes, and sensitivity to change), applicability to international comparative research, and susceptibility to bias and degree of technical expertise/support required to complete the instrument. For the assessment of the quality improvement system, the project will draw on the previous experience of the MARQuIS and ENQUAL instruments to assess hospital quality improvement systems [23,11]. Professional involvement will be assessed using a new instrument based on current work in the field [30-33]. Organisational culture will be assessed using existing measures, the selection of which will be informed by a recent and comprehensive review of measures [34]. Patient empowerment, understood as patients' contributions to quality management through which they are able to express their needs, present their concerns, devise strategies for involvement in decision-making and achieve political, social and cultural action to meet those needs, will be based on research previously performed in the MARQuIS and ENQuAL projects. Hospital governance and external pressure will be assessed using existing classifications [35,36,24].

#### Patient level measures

Patient level measures include clinical effectiveness, patient safety and patient involvement. We conceptualize measures of clinical effectiveness in two ways: firstly, to address care provision in line with evidence-base standards, and, secondly, to address outcomes of care [37]. For patient safety measures we refer to any measure that potentially prevents harm caused by errors of commission or omission [38]. In order to improve relevance to quality improvement and to comply with the criteria implicit in the definitions of clinical-practice guidelines, measures of clinical effectiveness and patient safety will be initially assessed in relation to discrete patient characteristics. Measures of clinical effectiveness and patient safety will be retrieved through audit of patient records, which is a common approach in epidemiological research, quality assessment and improvement and clinical research. Previous work on comparisons of coding, documentation and completeness of patient records in European countries will guide the logistics of record audit [39]. With regard to data extraction formulae, substantial work has been done on developing quality measures in Denmark, the USA and other countries and we will draw on existing work in order to refine and standardise hospital data, data transmission and performance measures in order to construct one robust, prioritised and standard quality measure set for hospitals. The need for risk-adjustment will be assessed on the basis of the final measures selected, and whether they are process or outcome measures.

Patient involvement is understood as the extent to which patients participate in decisions related to their condition (through informed consent, therapy plan or self-management) and contribute to organisational learning through their expert knowledge acquired during illness and hospitalisation [40,41]. Patient involvement will be assessed using a combination of existing patient surveys, drawing on existing measures of patient-centredness, shared-decision making and discharge preparation [42-46]. Instruments will be assessed for suitability (domain mapping) and discriminatory power. Measures will then undergo a process of backward and forward translation and cognitive testing. Psychometric analysis will address cultural differences and operational equivalence across countries [47].

In addition, we will collect administrative data for selected indicators. Since these data are readily available, are inexpensive to acquire, are computer readable and typically encompass large populations, they will be used for additional evaluation and validation studies. Concretely, we will collect disease-specific mortality rates (for acute myocardial infarction and stroke) and data on generic patient safety issues. These measures will be used for validation studies of the classification instrument for quality improvement systems. Additional work will address the potential for using administrative databases for quality improvement activities in the future [48].

### Data analysis

We envisage a series of statistical analyses with two main aims: (i) to validate the project constructs and (ii) to provide results for the four core objectives of the project. To address these two analytical aims, we will conduct any of four types of statistical analyses where appropriate: (a) descriptive, (b) data reduction and classification, (c) bivariate, and (d) multivariate analyses.

Descriptive statistics and graphical representations will be used to summarize the central tendencies, spread, frequencies or distributions of all relevant variables. Data reduction, classification techniques and psychometric methods will be employed to investigate the psychometric properties of questionnaires and other instruments used in measuring constructs relevant to the project such as organisational culture, professional involvement, and patient involvement. Before embarking on multivariate analyses, the project will conduct a series of bivariate analyses. As a prelude to the multivariate models, unadjusted regressions linking the relevant explanatory variables to their outcomes will also represent a type of bivariate analysis. The multivariate analyses will be conducted on two levels: hospital-level and multilevel. The hospital-level multivariate analysis specifies both explanatory variables and outcomes at the hospital-level. All analyses will adjust for relevant covariates as determined within the analytical framework, guided by background knowledge and theory [49]. The multilevel multivariate analysis refers to regression analysis of patient outcomes nested within hospitals, with adjustment for both patient-level and hospital-level covariates [50]. The multivariate regressions are by far the most important analyses needed for providing answers to the project's research objectives. The techniques of policy analysis [51,52] will be applied to the fourth research objective--identifying external factors influencing uptake of quality improvement activities.

We will introduce and apply a few sophisticated methodological elements which will be innovative for the field of performance and quality of care research. These innovative elements will constitute significant progress for health services research because they represent recent methodological advances from biostatistics, epidemiologic methodology, causality in artificial intelligence, and philosophy of science [53,54]. The first innovative element involves using directed acyclic graphs or causal diagrams to guide the specification of the abovementioned multivariate regressions which translate the analytical framework of the project [55]. The second innovative element involves extending the foregoing regressions which directly model the outcomes under study to modelling the explanatory variable (or exposure) as well, and then using the modeled version of the exposure to analyse the outcome in a second step [56]. The third innovative element is that we will further extend the outcome regression and exposure modeling by combining the two modeling approaches in the so-called doubly robust estimation [57,58]. Finally, given the wide variety of variables being conceptualised and measured in this project, we will conduct detailed sensitivity analysis or multiple bias analysis to assess the impact of the various sources of bias such as measurement error, (non-)response, uncontrolled confounding, and missing data on our results [59-61]. Missing data will be imputed using multiple imputation techniques [62]. All core analyses will be conducted with and without imputation for individual variables, including variables that will be imputed at the level measurement, that is, before aggregation to form hospital-level measures. The richer information contained in the multilevel datasets is expected to improve the multiple imputations.

### Analysis of policy implications

Based on the data analysis an appraisal scheme will be developed to guide hospitals in developing their quality improvement systems and to inform purchasers about the most effective quality improvement mechanisms. These appraisal schemes will differ in presentation, level of detail and focus. Their design will follow recent research on the development of tools to guide evidence-informed health policy making [63,64]. The guidance document for hospitals provides a more in-depth overview of the effectiveness of quality and safety strategies and how to integrate them at hospital and departmental level. The guidance document for commissioners/purchasers will aim at synthesising the main messages and identifying the core quality and safety strategies that should be in place at hospital and department level. It is expected that such criteria would be of use in subsequent contracting of hospital services.

### Ethics and confidentially issues

The project has been granted financial support and ethical approval from the European Commission. In addition, the ethics unit from the Department of Health in Catalonia, Spain, where the project lead is based, has confirmed the DUQuE project can be characterised as clinical audit (in contrast to human research) and as such does not require further ethical approval by the Catalan Ethical Committee for Clinical Research beyond that already granted by the European Commission in reviewing and approving the enclosed description of work.

This assessment is based on the distinction between clinical audit and human research introduced by the Council of Europe Draft Guide for Research Ethics Committee Members [65], according to which clinical audit is characterized by: a) the purpose of improving the quality of patient care in the local setting; b) measuring practice against standards; and c) doing nothing to patients which would not be part of routine practice. Other ethical committees have passed similar evaluations for research that is based on common quality improvement methods and that does not interfere with patient care in any way [66].

The project has developed strict privacy and confidentiality criteria for sampling of hospitals, professionals and patients, and for data collection, processing, analysis and reporting. These procedures are described in the project proposal approved by the European Commission and in complementary internal documents that specify information flow and technological issues around data processing.

### Investigators

The research team comprises experienced clinical, organisational and social science researchers, with experience in health services research, quality improvement research, organisational behaviour, survey design, statistical modelling, accreditation research, indicator development, external evaluation and clinical practice. Most researchers have previously been extensively involved in cross-national comparative quality improvement and health services research. Moreover, the researchers are affiliated with some of the main organisations driving quality improvement research in their respective countries and in Europe. In addition, the key research team will draw on the knowledge of a number of experts in dedicated content areas.

## Discussion

The results of the research should be relevant to multiple stakeholders, such as the European Commission and member states, healthcare institutions (hospitals and purchasing agencies). The results are also expected to contribute to advances in to methodological quality improvement research.

### Policy impact

At the policy level, as summarised in the previous section, considerable advances have been made in assessing quality improvement systems in the last years. However, an important question remains: what is the effect of quality improvement strategies and which combination of strategies work best? The objective of this project follows this question and aims at expanding previous research, taking into account the contextual variables of hospitals and patients' pathways at departmental level. This research would then be able to give guidance on a comprehensive set of strategies demonstrated to be effective whilst being sensitive to the context/country of a hospital. Having the unique advantage of basing DUQUE on leading existing research and the most recent contributions to the field, the following advances to the existing research literature will be made. Firstly, several innovative research projects are underway internationally, in particular in the USA and in Australia. We will link to these leading initiatives and apply the latest methods to European hospitals. Secondly, we are collaborating with existing international efforts to develop and validate performance indicators on quality and safety in health care. Thirdly, the results of several previous EU-funded projects are at this stage conceptually and empirically outdated [14,15,11]. We will advance existing work both conceptually, by exploring links between external pressure, quality systems and patient-level outcomes, and by collecting up-to-date data from a large sample of European hospitals. Fourthly, the MARQuIS project contributed to the research agenda by exploring the impact of hospital quality improvement systems on the uptake of specific quality and patient safety initiatives at the departmental level. DUQuE will further enhance, refine and systematise the various components (quality strategies and quality improvement systems) and will establish the nature of the relationship with process and outcome measures at patient level. Fifthly, the project is of high relevance in the context of the recent proposal for an EU directive on the application of patients' rights to cross-border health care [67]. Given that it is impossible to predict which hospitals will provide cross-border care in the future, all hospitals need to make sure that the appropriate quality and safety mechanisms are in place [68,69]. The DUQUE research project addresses this policy issue.

### Scientific contributions and methodological innovations

The proposed research project also makes distinctive contributions to improving quality and safety of care in the EU using an empirical approach. From a methodological perspective, the scientific contributions of the project are three-fold. Firstly, we will advance the validation of a number of relevant constructs relevant to advancing quality improvement systems in European hospitals. For example, we will select, adapt and build measures of organisational culture, professional involvement and patient empowerment, which in themselves can be used in further studies at national or European level. Secondly, by adding these measures to the organisational assessment, we will make relevant scientific contributions to understanding the interactions between these constructs and the hospitals' quality improvement systems. Thirdly, by including measures of external pressure and hospital governance on the one hand, and using patient-level outcome data on the other, we will be able to establish associations that exist between constructs that will result in our appraisal scheme for quality improvement systems. For the analysis of the large scale database we will include a number of methodological innovations described above.

## Conclusion

Research on quality in health care has over the last 30 years resulted in a substantial increase in knowledge of the measurement of quality, on variations in health care delivery, on the implementation of clinical practice guidelines based on best-evidence, on assessing patient satisfaction and experience and, more recently, on estimating the incidence of adverse events, which led to the patient safety movement. For several of these issues and based on scientific evidence, consensus exists, which has been summarised in appraisal schemes that facilitate synthesis of information, development of new tools and their application. Nevertheless, evidence of the effectiveness of organisational quality improvement systems has only more recently been accumulated. The DUQuE Research Project builds on the work developed to understand the impact of the implementation of different systems and aims to address their global effects through integration within an organisational quality improvement system.

## Competing interests

The authors declare that they have no competing interests.

## Authors' contributions

RS is the principal researcher of the study and OG is co-principal researcher. RS, OG, NK, CW, OA, AT and CB equally contributed to developing the research question, study design and implementation of the study protocol. OA was responsible for drafting the analysis plan. OG drafted the overall manuscript. All those listed as authors were responsible for reading, commenting upon, and approving the final manuscript.

## Pre-publication history

The pre-publication history for this paper can be accessed here:

http://www.biomedcentral.com/1472-6963/10/281/prepub
